# Gradual Increase of FcγRIIIa/CD16a Expression and Shift toward IFN-γ Secretion during Differentiation of CD56^dim^ Natural Killer Cells

**DOI:** 10.3389/fimmu.2017.01556

**Published:** 2017-11-20

**Authors:** Laurie Lajoie, Nicolas Congy-Jolivet, Armelle Bolzec, Gilles Thibault

**Affiliations:** ^1^CNRS UMR 7292, Génétique, Immunothérapie, Chimie et Cancer (GICC), Université François-Rabelais, Tours, France; ^2^Laboratoire d’Immunologie, Centre Hospitalier Régional Universitaire, Tours, France

**Keywords:** CD56^dim^ natural killer cells, FcγRIIIa/CD16a, degranulation, IFN-γ, killer Ig-like receptors, NKG2A, therapeutic monoclonal antibody

## Abstract

Natural killer (NK) cell effector functions include cytotoxicity and secretion of cytokines such as interferon-γ (IFN-γ). The immature CD56^bright^ subset of human NK cells lacks expression of FcγRIIIa/CD16a, one of the low-affinity immunoglobulin G receptors, or exhibits low-density expression (CD56^bright^CD16^−/dim^) and produces IFN-γ in response to cytokine stimulation, whereas the mature CD56^dim^CD16^+^ subset is the most cytotoxic one. A further differentiation/maturation of the latter subset according to the gradual loss of NKG2A and/or gain of KIR2DL (CD158a and CD158b) has been demonstrated and the ability to produce IFN-γ in response to activating receptor (AR) co-engagement is gradually acquired during terminal differentiation. In the course of flow cytometry analysis of CD56^dim^ NK cells, we noted a substantial intraindividual heterogeneity of expression of FcγRIIIa. FcγRIIIa is unique among ARs: it does not require the co-engagement of other ARs to induce substantial cytotoxicity or cytokine synthesis in CD56^dim^ cells. We, therefore, investigated whether individual differentiation/maturation of polyclonal CD56^dim^ NK cells defined by expression of NKG2A/KIR2DL is related to FcγRIIIa expression and to the heterogeneity of NK cell responses upon FcγRIIIa engagement. When we analyzed unstimulated CD56^dim^ cells by increasing level of FcγRIIIa expression, we found that the proportion of the more differentiated CD158a,h^+^ and/or CD158b,j^+^ cells and that of the less differentiated NKG2A^+^ cells gradually increased and decreased, respectively. FcγRIIIa engagement by using plate-bound murine anti-CD16 monoclonal antibody (mAb) or rituximab or trastuzumab (two therapeutic mAbs), resulted in donor-dependent partial segregation of IFN-γ-producing and/or degranulating CD56^dim^ cells. Importantly, the proportion of CD158a,h/b,j^+^ cells and that of NKG2A^+^ cells was increased and decreased, respectively, IFN-γ-producing cells, whereas these proportions were poorly modified in degranulating cells. Similar results were observed after engagement of ARs by a combination of mAbs targeting NKG2D, NKp30, NKp46, and 2B4. Thus, the gradual increase of FcγRIIIa expression is an important feature of the differentiation/maturation of CD56^dim^ cells and this differentiation/maturation is associated with a shift in functionality toward IFN-γ secretion observed upon both FcγRIIIa-dependent and FcγRIIIa-independent stimulation. The functional heterogeneity related to the differentiation/maturation of CD56^dim^ NK cells could be involved in the variability of the clinical responses observed in patients treated with therapeutic mAbs.

## Introduction

Natural killer (NK) cell effector functions include natural cytotoxicity, antibody-dependent cell-mediated cytotoxicity (ADCC), and secretion of cytokines such as interferon γ (IFN-γ) and tumor necrosis factor α. Two subsets of human NK cells depending on the density of CD56 expression have been identified. CD56^dim^CD16^+^CD3^−^ cells usually account for more than 90% of the NK cells in peripheral blood, whereas CD56^bright^CD16^dim/−^CD3^−^ cells are more common in lymphoid organs (1–3). The major effector function of the former cells may be cytotoxicity, whereas the latter may act mainly *via* cytokine secretion (2). However, most NK cells that are cytotoxic and/or produce IFN-γ on stimulation with different types of target cells (4–7), including K562 and antibody-coated target cells (5), belong to the CD56^dim^ subset. In contrast, NK cells that readily respond to cytokines such as IL-12 and IL-15, belong to the CD56^bright^ NK cell subset (2, 5). CD56^dim^ and CD56^bright^ NK cells may be more appropriately defined as “target cell-responsive” and “cytokine-responsive,” respectively (5).

The regulation of NK cell functions depends on a very fine balance between signals mediated by activating receptors (ARs) and inhibitory receptors (IRs) (6, 8). ARs mainly include the natural cytotoxicity receptors (NKp46/CD335, NKp44/CD336, NKp30/CD337), NKG2D/CD314, 2B4/CD244, and FcγRIIIa/CD16a, one of the low-affinity immunoglobulin G (IgG) receptors involved in ADCC (8, 9). IRs mainly include the C-type lectin NKG2A/CD94 heterodimer receptor, which recognizes human leukocyte antigen (HLA)-E molecules and killer Ig-like receptors (KIR) such as KIR2DL1 (CD158a), specific to the HLA-C group C2 allotype, and KIR2DL2/3 (CD158b), specific to the HLA-C group C1 allotype (10, 11).

According to the process referred to as “education or licensing” of NK cells, acquisition of functional responses depends on the engagement of IRs with self-ligands during their development (5, 12, 13). Remarkably, the vast phenotypic diversity in the human NK cell repertoire is related to the broad range of possible combinations of phenotypes on a single cell from a given donor. Thus, all NKG2A and KIR expression patterns are represented, including NK cells lacking IRs for self, which remain hyporesponsive (5, 12, 13).

Activating receptors involved in natural cytotoxicity such as NCR, NKG2D, and 2B4 can signal independently, but functional responses, including cytotoxicity and cytokine synthesis, require a combination of signals resulting from two or more interactions between different receptor–ligand pairs (14–16). By contrast, the FcγRIIIa receptor is unique in its ability to induce both responses without additional signal provided by co-engagement of other ARs (14–16). A partial dichotomy between IFN-γ-producing and degranulating NK cells upon FcγRIIIa engagement by anti-CD16-sensitized P815 cells (5) or by CD20^+^ cells opsonized with the therapeutic anti-CD20 monoclonal antibody (mAbs) rituximab (RTX) or obinituzumab (17) was previously reported. How a given AR induces different functional responses within the polyclonal NK cells of a given donor was not specifically discussed.

A stepwise differentiation/maturation of NK cells from the immature CD56^bright^CD16^−^ (NKG2A^++^KIR^−^) cells through the intermediate CD56^bright^CD16^dim^ stage to the mature CD56^dim^CD16^+^ (NKG2A^±^KIR^±^) population is usually admitted (18–21). A further differentiation/maturation of the CD56^dim^CD16^+^ subset according to the gradual loss of NKG2A and CD62L and/or the gradual gain of KIRs and CD57 (21–26) has been demonstrated, supporting the concept of a continuous process starting from CD56^bright^NKG2A^++^KIR^−^CD62L^+^CD57^−^cells and ending with the CD56^dim^NKG2A^−^KIR^+^CD62L^−^CD57^+^ phenotype. This phenotype change is associated with a shift in functionality from cytotoxicity/degranulation toward IFN and TNF secretion in response to ARs stimulation (27). While this effect is most strikingly observed in CD57^+^ NK cells, it has also been observed when comparing NKG2A^+^KIR^−^ with NKG2A^−^KIR^+^ NK cells stimulated by target cells in the context of NK cell transplantation (7). In addition, it has been shown that activation of CD56^dim^ NK cells results in the down-modulation of FcγRIIIa by ADAM17-mediated shedding or internalization (16, 28), resulting in the presence of CD56^dim^CD16^−^ cells in peripheral blood. Finally, the *FCGR3A* gene, which encodes FcγRIIIa, displays a functional allelic dimorphism generating allotypes with either a phenylalanine (F) or a valine (V) at amino acid position 158 (29, 30). The V158F polymorphism of FcγRIIIA, which is associated with higher therapeutic response to RTX (31–33) or to the anti-ErB-2 mAb trastuzumab (TTZ) used in brain cancer (34), has also been related to interindividual variations in FcγRIIIA expression (35, 36), although this is not confirmed (9, 37). In the course of flow cytometry (FCM) analysis of human NK cells, we also noted a substantial intraindividual heterogeneity of FcγRIIIa expression on CD56^dim^ NK cells in accordance with a recent study, reporting the presence of an individualized subset of CD56^dim^ cells expressing low level of CD16 in human peripheral blood (38).

Here, we aimed to investigate whether individual differentiation/maturation of polyclonal CD56^dim^ NK cells defined by expression of NKG2A/KIR2DL is related to FcγRIIIa expression and to the heterogeneity of NK cell responses upon FcγRIIIa engagement. We used multi-color FCM (25, 39) to simultaneously evaluate the individual expression of NKG2A, CD158a, and CD158b on CD56^dim^ NK cells and (1) the level of FcγRIIIa expression on unstimulated cells and (2) the degranulation and IFN-γ production in response to FcγRIIIa engagement by plate-bound anti-CD16 mAb, or IgG1 therapeutic mAbs RTX and TTZ.

## Materials and Methods

### Monoclonal Antibodies

The following mAbs were used: unconjugated anti-CD16 (clone 3G8)/IgG1; FITC- and APC-Alexa Fluor 750-conjugated anti-CD16 (clone 3G8)/IgG1 (final dilution 1:50 and 1:200, respectively); PE- and APC-Alexa Fluor 700-conjugated anti-CD56 (clone N901)/IgG1 (final dilution 1:50); APC-conjugated anti-NKG2A (clone Z199)/IgG1 (final dilution 1:50); PE-conjugated anti-IFNγ (clone 45.15)/IgG1 (final dilution 1:50); PE-conjugated anti-CD158a (clone EB6.B), which recognizes also the activating isoform CD158h/IgG1 (final dilution 1:50); and PeCy5.5-conjugated anti-CD158b (clone GL183, which recognizes also the activating isoform CD158j)/IgG1 (final dilution 1:50), all from Beckman Coulter (Villepinte, France). Antibodies targeting NKG2D/CD314 (clone 1D11), NKp30/CD335 (clone 4D12), NKp46/CD335 (clone 9E2), 2B4/CD244 (clone 2-69), and isotype control, PECy-7-conjugated anti-IFNγ (clone B27)/IgG1 (final dilution 1:100), FITC- and PeCy-5-conjugated anti-CD107a (clone H4A3)/IgG1 (final dilution 1:50) and their isotype controls were from BD Biosciences (Le Pont de Claix, France). RTX and TTZ were kindly provided by Dr. Tournamille (CHRU de Tours, France).

### NK-Cell Isolation

Peripheral blood mononuclear cells (PBMCs) were exclusively obtained from the blood of healthy volunteers (i.e., blood donors from the Etablissement Français du Sang Centre-Atlantique, who had given their written informed consent) according to institutional research protection guidelines (Agreement No IMMUNOUMR6239/37/12/01) after centrifugation over lymphocyte separation medium (Eurobio, les Ulis—Courtaboeuf, France). NK cells were isolated by using the NK cell Isolation Kit MACS (Miltenyi Biotec, Paris, France). The purity was consistently ≥95%.

### Coating Culture Plates with mAbs

NUNC Maxisorp culture plates (Fisher Labosi, Elancourt, France) were sensitized or not for 12 h at 4°C with 5 µg/mL or indicated concentrations of anti-CD16mAb, or with 5 µg/mL of RTX (9) or TTZ or with 5 µg/mL of a combination of mAbs targeting NKG2D/CD314, NKp30/CD335, NKp46/CD335, 2B4/CD244 (16). After three washes with phosphate-buffered saline (PBS) TWEEN solution (45 µL Tween 20 from Sigma Aldrich in 100 mL PBS), plates were saturated for 30 min with bovine serum albumin 1% (Sigma Aldrich, Saint Quentin Fallavier, France), then washed three times with PBS Tween.

### *In Vitro* Stimulation of NK Cells, Analysis of CD16 and Inhibitory Receptor Expression and Functional Responses

In total, 100 µL freshly isolated NK cells (1 × 10^5^) were plated on unsensitized or sensitized plates and incubated at 37°C in 5% CO_2_ humidified air (usually 4 h; from 1 to 20 h in kinetics experiments) in the presence of anti-CD107amAb and 0.1 µg/mL BD GolgiPlug containing Brefeldin A (BD Biosciences). When indicated, cells were stained with anti-CD16, anti-CD56 anti-NKG2A, anti-CD158a,h, and anti-CD158b,j mAbs for 30 min at 4°C. Cells were then fixed and permeabilized by using the BD Cytofix/cytoperm Plus Kit (BD Biosciences) and stained for intracellular IFNγ with anti-IFNγ mAb for 30 min at 4°C.

### FCM Analysis

Functional responses and phenotypes of cell subsets were analyzed by FCM. All FCM analyses were performed with a Gallios flow Cytometer and Kaluza 1.3 software (Beckman Coulter).

### Statistics

Comparison of proportions of cells expressing each IR to all CD56^dim^ NK cells were analyzed using the repeated measures ANOVA, bonferroni multiple comparisons test with GraphPad Prism 5 software. *P* < 0.05 was considered statistically significant.

## Results

### FcγRIIIa Expression Gradually Increases during CD56^dim^ NK Cell Differentiation/Maturation Defined by NKG2A and KIRs Expression

**Figure 1:**
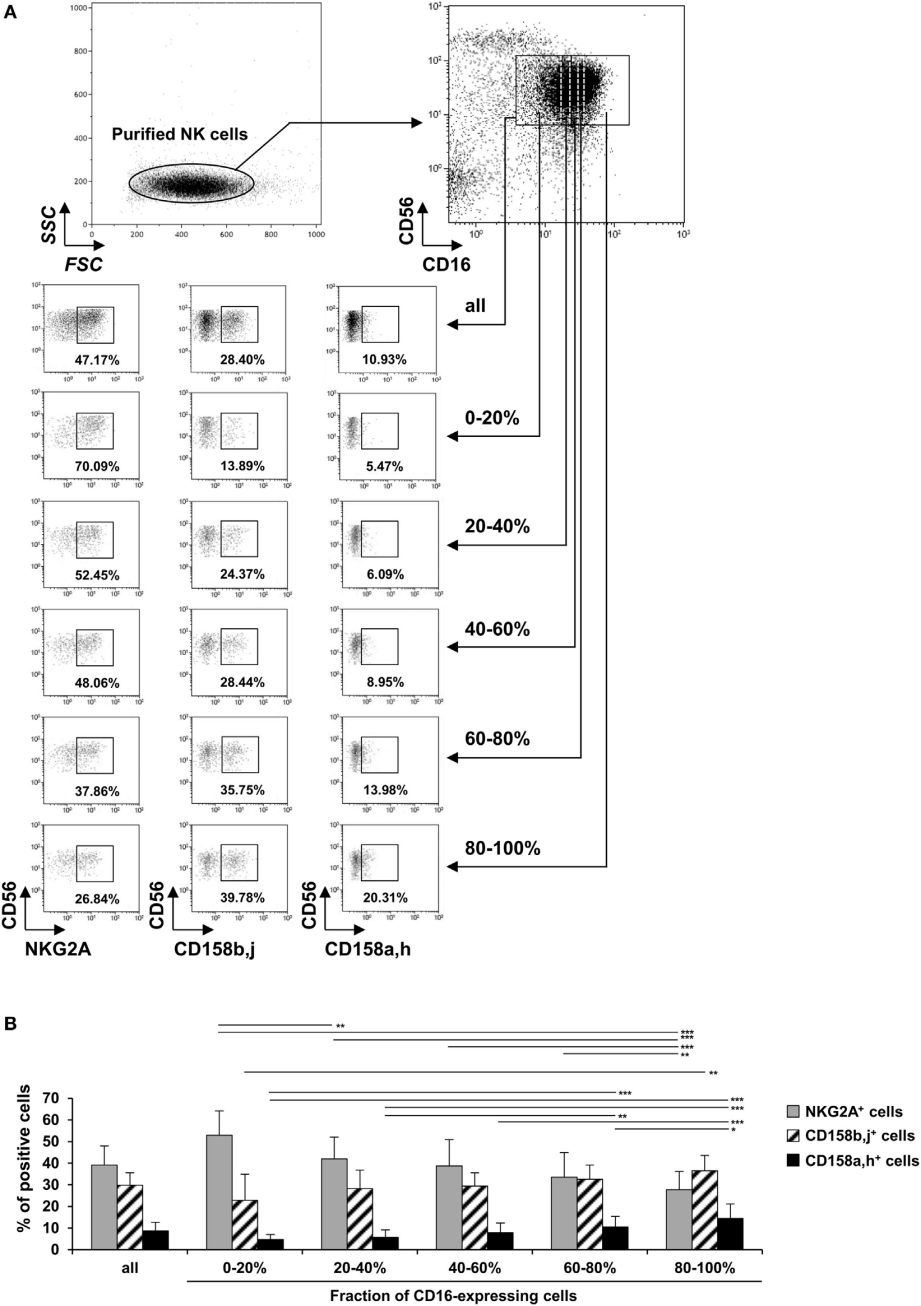
Expression of NKG2A, CD158b, and CD158a on CD56^dim^ natural killer (NK) cells by expression of FcγRIIIa. Freshly isolated NK cells were stained with fluorescent anti-CD56, anti-CD16, anti-NKG2A, anti-CD158a,h, and anti-CD158b,j monoclonal antibodies and analyzed by flow cytometry (FCM). **(A)** One representative experiment showing the gating strategy and results from one donor. The FcγRIIIa-expressing CD56^dim^ cells were arbitrarily divided into five equal parts by level of FcγRIIIa assessed by FCM and the percentage of cells expressing NKG2A, CD158b,j, and CD158a,h was analyzed in each part. **(B)** Percentages of NKG2A^+^, CD158b,j^+^, and CD158a,h^+^ cells among all CD16^+^CD56^dim^ NK cells and each fraction of CD16-expressing cells. Data are mean ± SD. *n* = 7. *P < 0.05, **P < 0.01, ***P < 0.001.

We first investigated whether the FcγRIIIa expression could be related to the differentiation/maturation stage of CD56^dim^ NK cells. These cells gradually lose NKG2A and acquire KIRs during their differentiation/maturation (21–24). Hence, we compared the proportion of total NKG2A^+^, total CD158b,j^+^ and total CD158a,h^+^ cells by FcγRIIIa expression on CD56^dim^ NK cells. We and others have previously shown that stimulating NK cells results in ADAM-17-dependent FcγRIIIa down-modulation (16, 28). Therefore, FcγRIIIa expression was evaluated on unstimulated NK cells. The FcγRIIIa-expressing CD56^dim^ cells of each donor (*n* = 7) were arbitrarily divided into five equal parts by level of FcγRIIIa assessed by FCM (Figure 1A right upper panel; Figure S1 in Supplementary Material). We observed a substantial interindividual variation of FcγRIIIa staining, which could be related to the V158F polymorphism of FcγRIIIa, as previously described (9, 35–37). Therefore, the setting of the five gates used to divide the FcγRIIIa-expressing cells, differs from one donor to another (Figure S1 in Supplementary Material). The proportion of cells expressing each IR was then analyzed in each part (gating strategy shown in Figure 1A). In all donors tested, the proportion of NKG2A^+^ NK cells decreased with increasing level of FcγRIIIa on CD56^dim^ cells, but the proportion of CD158b,j^+^ and CD158a,h^+^ cells increased (Figure 1B). Thus, NKG2A^+^ cells were 2.5 times more numerous, on average, than CD158b,j^+^ cells among the 20% of CD56^dim^ cells expressing the lower level of FcγRIIIa, whereas CD158b,j^+^ cells were the majority among the 20% of CD56^dim^ cells expressing the higher level of FcγRIIIa. Results were similar when comparing cells expressing a single IR (i.e., NKG2A^+^CD158a,h^−^CD158b,j^−^, NKG2A^−^CD158a,h^+^CD158b,j^−^, and NKG2A^−^CD158a,h^−^CD158b,j^+^ cells; data not shown). Moreover, we performed the reverse analysis, comparing the mean expression of FcγRIIIa on total NKG2A^+^, CD158b,j^+^, and CD158a,h^+^ CD56^dim^ NK cells, from the same seven donors. As expected, we found that the FcγRIIIa level [expressed as mean fluorescence intensity (MFI)] on NK cells from each donor was significantly associated with the IR coexpressed, in the order of CD158a,h^+^ ≥ CD158b,j^+^ > NKG2A^+^ cells. Results were again similar when comparing cells expressing a single IR (data not shown).

### IFN-γ-Producing CD56^dim^ NK Cells Partially Segregate from Degranulating Cells in Response to FcγRIIIa Engagement

**Figure 2:**
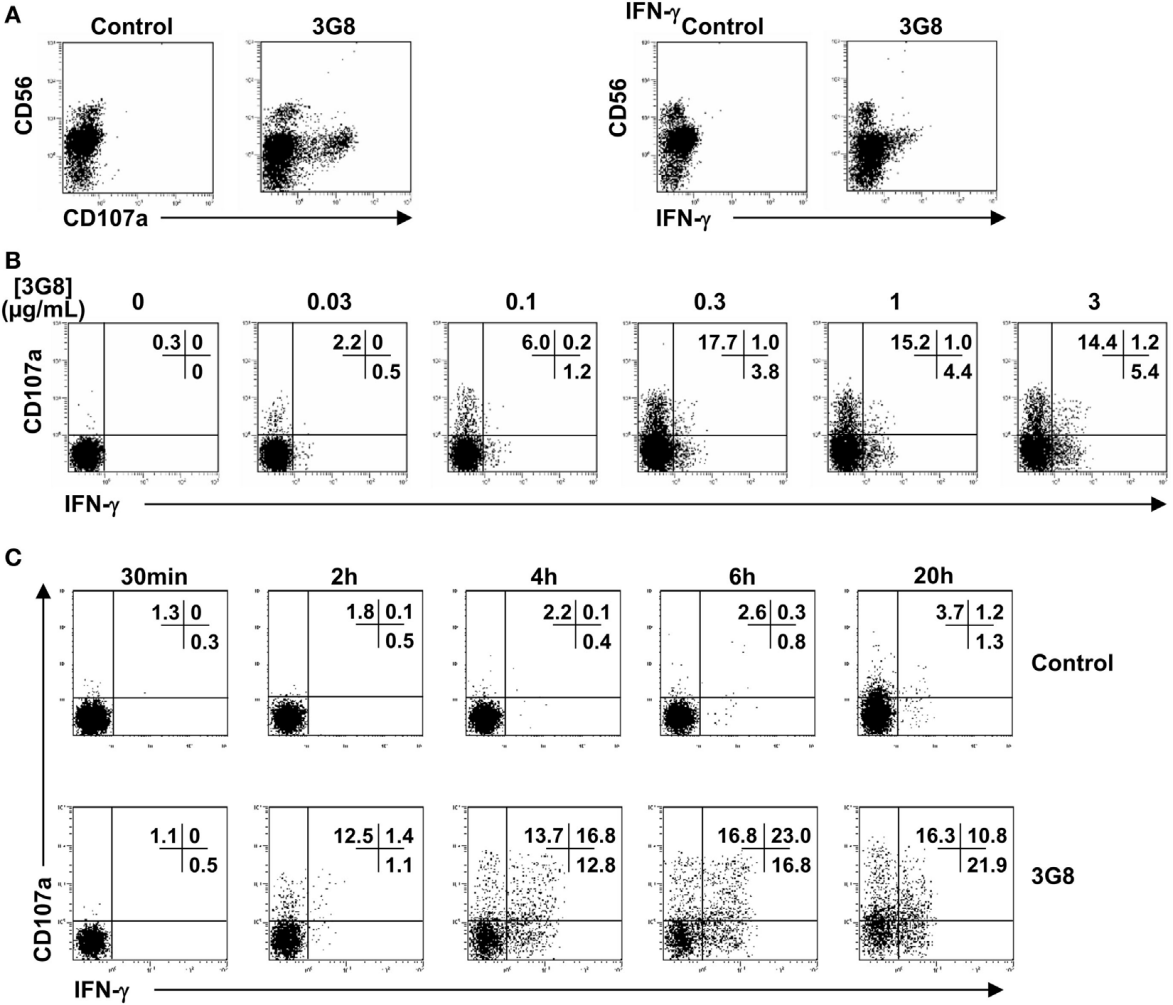
Degranulation and IFN-γ synthesis by CD56^dim^ NK cells in response to FcγRIIIa engagement by plate-bound anti-CD16 monoclonal antibody (mAb). Culture plates were sensitized overnight with a saturating concentration 5 μg/mL **(A,C)** or increasing concentrations **(B)** of anti-CD16 3G8 mAb. Freshly isolated natural killer (NK) cells were then incubated for 4 h **(A,B)** or for the times indicated **(C)** on coated plates, in the presence of anti-CD107a mAb and brefeldin A. Cells were then stained with anti-CD56 mAb. Fixed and permeabilized NK cells were stained for intracellular IFN-γ expression and analyzed by FCM. Results are from one representative of three independent experiments (obtained with NK cells from three donors).

To analyze the profile of NK cell functions in response to a single condition of stimulation (i.e., FcγRIIIa engagement), purified polyclonal NK cells were cell-free stimulated by plate-bound anti-CD16 (clone 3G8) mAb. As expected, the responding cells (Figure 2A) were mainly (CD107a^+^ cells) or exclusively (IFN-γ^+^ cells) CD56^dim^ cells. We previously showed that the adsorption of 3G8 mAb on the plates plateaued at 1 µg/mL (9). Here, we show that stimulated NK cells showed concentration-dependent CD107a expression and IFN-γ synthesis. Both responses were detected at 0.03 µg/mL and plateaued at 0.3–1 µg/mL (Figure 2B). More importantly, in our restricted condition of stimulation, we found a partial functional segregation in the responding NK cells: CD107a^−^IFN-γ^+^, CD107a^+^IFN-γ^−^, and CD107a^+^IFN-γ^+^subsets were detected in polyclonal NK cells from all donors tested. After 4 h of stimulation, most of the responding cells thus exhibited a single functional response (i.e., degranulation or IFN-γ production), although some cells exhibited both responses. Similar results were obtained when cells were stimulated with plate-bound RTX or TTZ (data not shown).

Degranulation is much faster than cytokine release (6). The partial dichotomy of the FcγRIIIa-dependent functional response on CD56^dim^ NK cells (Figure 2B) could, therefore, be explained by cells that degranulate and only produce cytokines after a further period of activation. To investigate the dynamics of the different NK responses, we examined the response of NK cells stimulated by a saturating concentration of plate-bound 3G8 mAb between 30 min and 20 h. We detected degranulation and IFN-γ synthesis after 2 h (Figure 2C). However, the proportion of CD107a^−^IFN-γ^+^, CD107a^+^IFN-γ^−^, and CD107a^+^IFN-γ^+^cells was still similar after 4 and 6 h. Moreover, the three subsets were still observed after 20 h of stimulation. Therefore, the partial segregation of IFN-γ-producing and degranulating cells within the polyclonal NK cells did not result from differences in the kinetics of these responses.

### Relative Proportion of IFN-γ-Producing and Degranulating CD56^dim^ NK Cells upon FcγRIIIa Engagement Is Donor-Dependent

**Figure 3:**
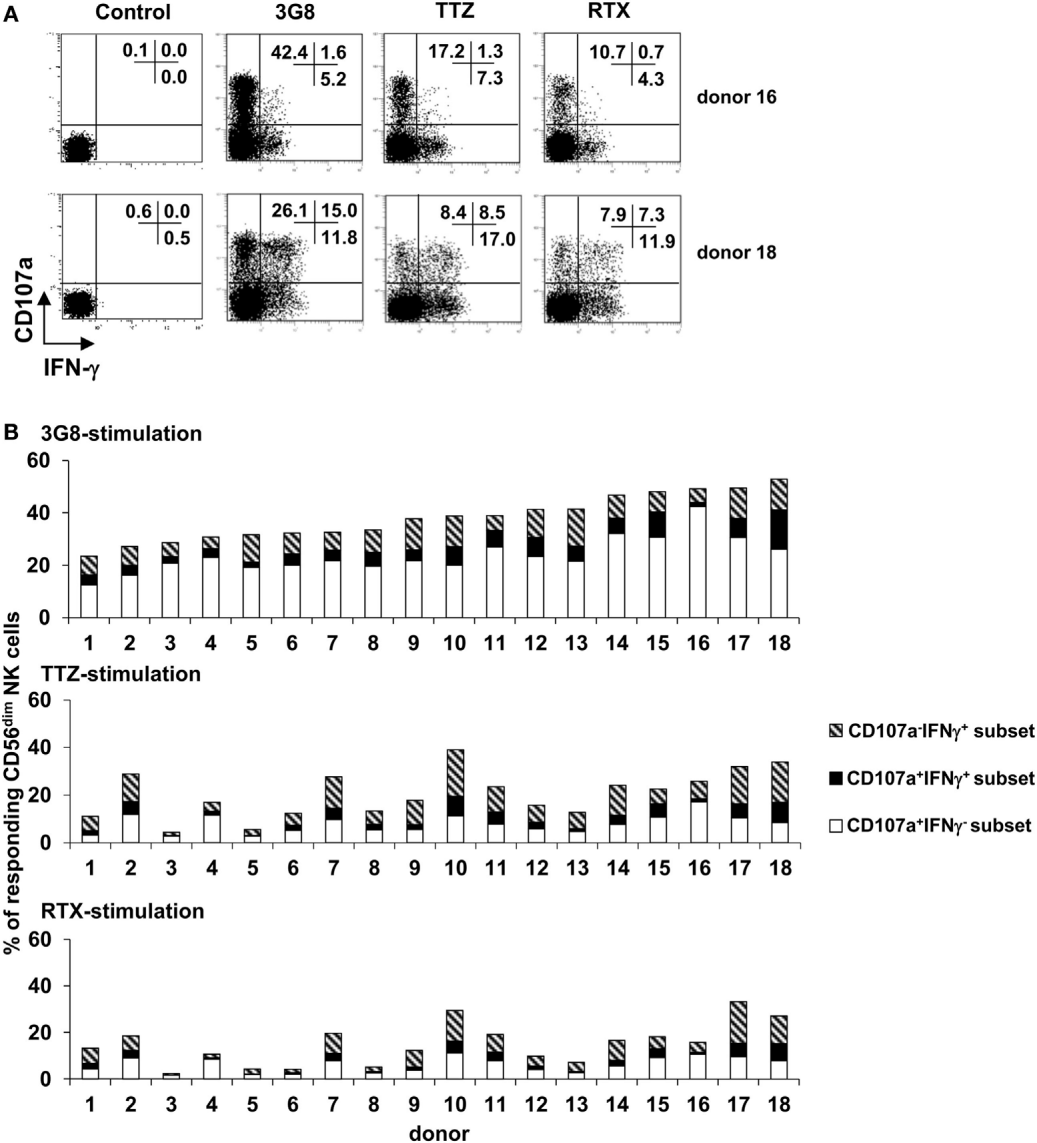
Inter-individual variation in the percentage of degranulating and/or IFN-γ-producing CD56^dim^ natural killer (NK) cells upon FcγRIIIA engagement. Culture plates were sensitized overnight without or with a saturating concentration of anti-CD16 3G8 monoclonal antibody (mAb) or trastuzumab (TTZ) or RTX. Freshly isolated NK cells were then stimulated as described for Figure 2A. **(A)** The proportion of CD107a^+^IFN-γ^−^, CD107a^−^IFN-γ^+^, and CD107a^+^IFN-γ^+^ NK cells was evaluated by flow cytometry. Plots are from one donor (among 18) with high proportion of degranulating cells (donor 16) and one donor with high proportion of IFN-γ-producing cells (donor 18). **(B)** The proportion of degranulating cells (white bars), IFN-γ-producing cells (striped bars), and cells exhibiting both responses (black bars) was evaluated in the 18 donors as shown in **(A)** upon stimulation with 3G8 mAb (upper panel), TTZ (middle panel), or RTX (lower panel). Donors were arbitrarily ranked according to the percentage of total responding cells upon stimulation by 3G8 mAb.

We then investigated whether the ability of polyclonal NK cells to degranulate and/or produce IFN-γ might fluctuate quantitatively by donor. Purified polyclonal NK cells from 26 healthy donors were stimulated with 3G8 mAb, as described above, or with plate-bound TTZ or RTX (18 of the 26 donors) (Figure 3A: FCM plots obtained in 2 donors. Figure 3B: histogram representation of results obtained in the 18 donors). As expected, we observed substantial interindividual variations in the percentage of responding cells (i.e., degranulating and/or IFN-γ-producing cells) whatever the stimulus. It is of note that the interindividual ranking of responding cells observed after 3G8 stimulation substantially differs from that observed after TTZ or RTX simulation. CD107a^+^IFN-γ^−^ cells largely predominated (61.1 ± 13.9%), followed by CD107a^−^IFN-γ^+^ cells (27.1 ± 14.2%) and double-positive CD107a^+^IFN-γ^+^cells (11.8 ± 5.9%) after stimulation with 3G8, but this ranking was not systematically observed after stimulation with TTZ or RTX. Indeed, the percentage of degranulating cells was substantially higher upon stimulation with 3G8 (Figure 3B upper panel) than with TTZ (Figure 3B middle panel) or RTX (Figure 3B lower panel), whatever the donor. Importantly, we also observed substantial interindividual variability in the relative proportion of the three subsets [for instance, the majority of responding cells were CD107a^+^IFN-γ^−^ in donor 4, 15, and 16 whatever the stimulus, whereas CD107a^−^IFN-γ^+^ and/or CD107a^+^IFN-γ^+^ were more frequent in donor 10, 14, 17, and 18 (especially after TTZ or RTX stimulation)]. Interestingly, a similar pattern (particularly the proportion of IFN-γ-producing cells) was observed in a given donor when cells were stimulated with 3G8, RTX, or TTZ. In accordance, we found no correlation between the proportion of CD107a^+^IFN-γ^−^ and CD107a^−^IFN-γ^+^ NK cells obtained from the 26 donors after 3G8 stimulation (*R*^2^ = 0.02) (data not shown). The results were similar when examining the correlation between all degranulating cells (CD107a^+^IFN-γ^−^ and CD107a^+^IFN-γ^+^) and all IFN-γ-producing cells (CD107a^−^IFN-γ^+^ and CD107a^+^IFN-γ^+^). Thus, the profile of NK-cell functional response to FcγRIIIa engagement by 3G8 mAb, RTX, or TTZ was highly donor-dependent.

### CD56^dim^ NK Cells Producing IFN-γ upon FcγRIIIa Engagement Are Mainly Differentiated NKG2A^−^KIR^+^ Cells

**Figure 4:**
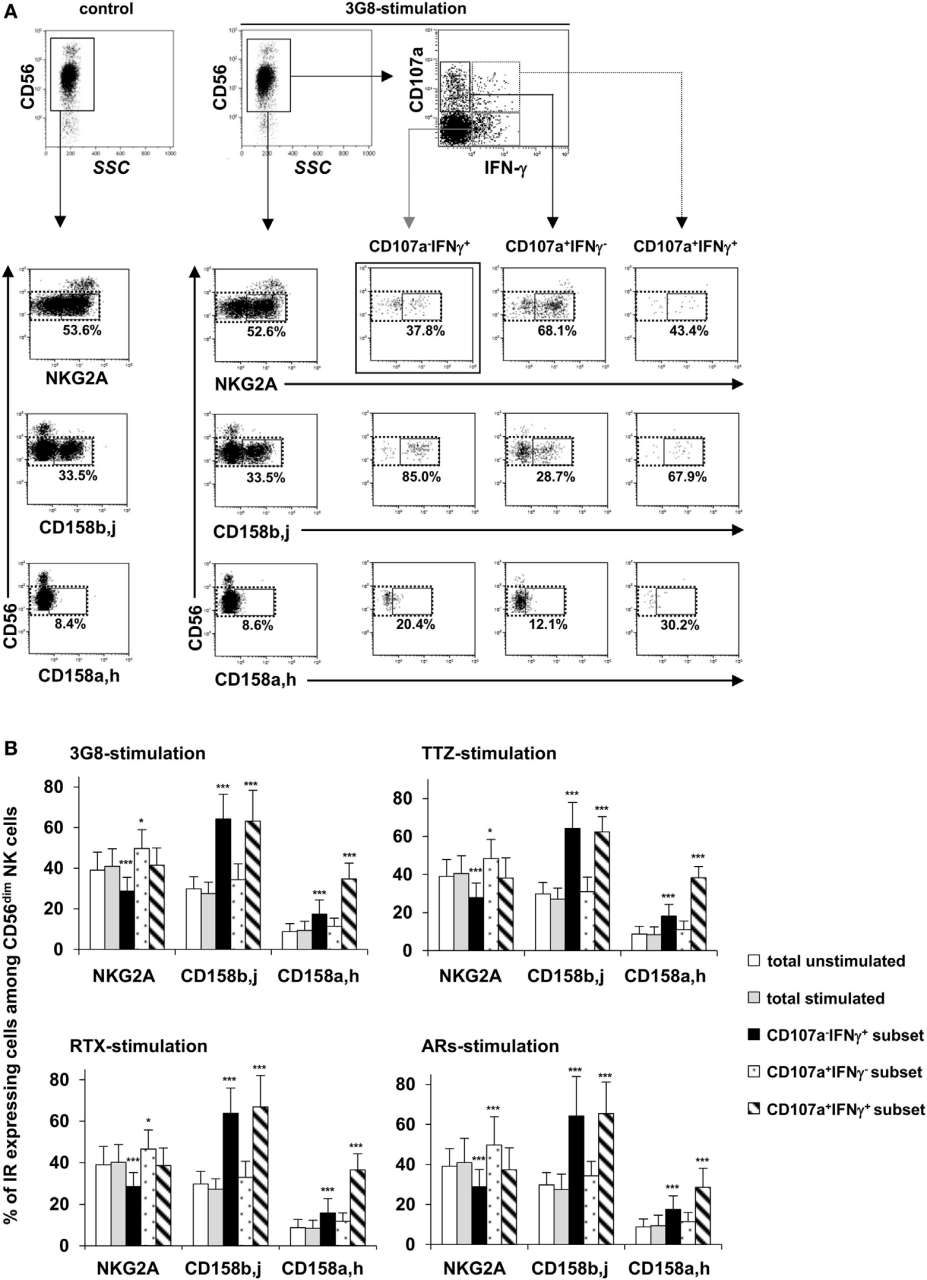
Proportion of NKG2A^+^, CD158b^+^, and CD158a^+^ cells among degranulating and/or IFN-γ-producing CD56^dim^ natural killer (NK) cells in response to FcγRIIIA engagement by plate-bound anti-CD16 monoclonal antibody (mAb), trastuzumab (TTZ), or RTX or in response to engagement of ARs involved in natural cytotoxicity. Culture plates were sensitized overnight without or with a saturating concentration of anti-CD16 3G8 mAb, TTZ, RTX, or a combination of mAbs targeting NKG2D, NKp30, NKp46, and 2B4 and freshly isolated NK cells were stimulated as described for Figure 2A. The proportion of NKG2A^+^, CD158b,j^+^, and CD158a,h^+^ cells was evaluated by flow cytometry on total unstimulated and stimulated NK cells and on CD107a^−^IFN-γ^+^, CD107a^+^IFN-γ^−^, and CD107a^+^IFN-γ^+^subsets (observed after stimulation) from seven donors. The percentage of NKG2A^+^, CD158b,j^+^, and CD158a,h^+^ cells among CD56^dim^ NK cells was calculated by dividing the number of cells within the CD16^dim^ IR + gate (solid lines) by the number of cells within the CD16^dim^ gate (dotted line). **(A)** One representative experiment showing the gating strategy and results from one donor. The percentages indicate the proportion of IR^+^CD56^dim^ NK cells among the total CD56^dim^ NK cell population. **(B)** Proportion of CD56^dim^ NK cell subsets expressing inhibitory receptors (NKG2A, CD158b,j, and/or CD158a,h) among total unstimulated CD56^dim^ NK cells (white bars), total stimulated CD56^dim^ NK cells (gray bars), and within each subset of responding cells [i.e., CD107a^−^IFN-γ^+^cells (black bars), CD107a^+^IFN-γ^−^cells (dotted bars), and CD107a^+^IFN-γ^+^cells (striped bars)] upon stimulation with anti-CD16 3G8 mAb (upper left panel), TTZ (upper right panel), RTX (lower left panel) or mAbs targeting NKG2D, NKp30, NKp46, and 2B4 (lower right panel). Data are mean ± SD. *P < 0.05, ***P < 0.001.

We then considered whether the degranulation and/or IFN-γ production induced by FcγRIIIa engagement could be related to the differentiation/maturation stage of individual NK cells. We, therefore, compared the proportion of NKG2A^+^, CD158a,h^+^, and CD158b,j^+^ cells among total CD56^dim^ NK cells and among the CD107a^−^IFN-γ^+^, CD107a^+^IFN-γ^−^, and CD107a^+^IFN-γ^+^ subsets (gating strategy is shown in Figure 4A) obtained on stimulation with plate-bound anti-CD16 3G8 mAb, TTZ, or RTX. First, the proportion of NK cells expressing each IR was as expected, unchanged after FcγRIIIa engagement by plate-bound anti-CD16 3G8 mAb (Figure 4B left upper panel), TTZ (Figure 4B right upper panel), or RTX (Figure 4B left lower panel). Second, the proportion of NKG2A^+^ cells in CD107a^−^IFN-γ^+^ and CD107a^+^IFN-γ^−^ subsets of CD56^dim^ NK cells was decreased and increased, respectively, whereas it was unmodified in the CD107a^+^IFN-γ^+^subset (Figure 4B). Third, the proportion of CD158b,j^+^ and CD158a,h^+^ cells in IFN-γ-producing cells (CD107a^−^IFN-γ^+^ and CD107a^+^IFN-γ^+^) was greatly increased, whereas it was weakly but not significantly increased in the CD107a^+^IFN-γ^−^ subset. Thus, NKG2A^+^ cells were about 1.4-times more numerous, on average, than CD158b,j^+^ cells among total unstimulated or stimulated CD56^dim^ NK cells. This ratio was unchanged among degranulating cells but was inverted (about 2.0-times more CD158b,j^+^ cells than NKG2A^+^ cells) among IFN-γ-producing cells. Importantly, the results obtained on stimulating NK cells with plate-bound TTZ or RTX were similar to those obtained on stimulation with plate-bound 3G8 mAb (compare Figure 4B upper and lower left panels and upper right panel). In addition, NKG2A and CD158b,j showed slightly increased expression (MFI) on CD56^dim^CD107a^+^IFN-γ^−^ and CD56^dim^CD107a^−^IFN-γ^+^ cells, respectively (data not shown). Finally, we wondered whether these results were unique to FcγRIIIa-dependent stimulation or also apply to engagement of other ARs. Therefore, NK cells from the same donors were incubated in plates sensitized by a combination of mAbs targeting NKG2D, NKp30, NKp46, and 2B4 (i.e., in the absence of FcγRIIIa engagement), as previously described (16). Results (Figure 4B, lower right panel) were similar to those observed in response to FcγRIIIa engagement. Thus, the shift toward IFN-γ secretion associated with the gain of KIRs and the loss of NKG2A was similarly observed in response to ARs involved in natural cytotoxicity and in response to FcγRIIIa engagement by either anti-CD16 mAb or by the Fc portion of therapeutic mAbs.

## Discussion

In this study, we show that within a given individual a gradual increase of FcγRIIIa expression is associated with the differentiation/maturation of CD56^dim^ NK cells from NKG2A^+^CD158a,h/b,j^−^ toward NKG2A^−^CD158/a,h/b,j^+^. We show, in addition, that FcγRIIIa engagement by using a murine anti-CD16 mAb or by the Fc portion of human therapeutic mAbs resulted in donor-dependent partial functional segregation of IFN-γ-producing and/or degranulating CD56^dim^ cells. Importantly, the proportion of CD158a,h/b,j^+^ cells and that of NKG2A^+^ cells was increased and decreased, respectively, in IFN-γ-producing cells, whereas the frequency of CD158a,h^+^, CD158b,j^+^, and NKG2A^+^ cells proportions were poorly modified in degranulating cells. Similar results were observed after engagement of ARs involved in natural cytotoxicity. Our results further support the notion of a continuous differentiation/maturation of CD56^dim^ NK cells defined phenotypically by a gradual increase of FcγRIIIa expression and associated with a shift in functionality toward IFN-γ secretion observed upon both FcγRIIIa-independent and FcγRIIIa-dependent stimulation.

The level of FcγRIIIa expression on NK cells has been shown to depend on several factors. For instance, we and others have demonstrated that the activation of CD56^dim^ NK cells results in the down-modulation of FcγRIIIa by ADAM17-mediated shedding or internalization (16, 25). Moreover, interindividual variations in FcγRIIIA expression related to the V158F polymorphism of FcγRIIIA has been reported (35, 36), although this is not confirmed (9, 37). Our results are unrelated to these observations. Indeed, we evaluated the correlation of FcγRIIIa and IR expression on unstimulated cells and compared cells of given individuals, excluding any effect of FcγRIIIa polymorphism. Variation of FcγRIIIa expression has been originally related to the existence of different subsets or maturation stages of NK cells defined by the level of CD56 expression: CD56^bright^ cells lack FcγRIIIa expression or exhibit low-density expression, whereas most CD56^dim^ cells are considered to express high level of FcγRIIIa (1, 2, 40). Thus, the relationship between expression of FcγRIIIa and the diffenciation/maturation of the latter cells has been sparsely studied. The differentiation/maturation of CD56^dim^ NK cells is associated with gain (KIRs and CD57) and loss (NKG2A and CD62L) of protein expression on the cell surface (21–26). On the one hand, Amand et al. have recently described a new subset of CD56^dim^ NK cells with low level of FcγRIIIa (38). Phenotypically, the new subset contained a high percentage of relatively immature cells, as reflected by a significantly stronger representation of NKG2A^+^ and CD57^−^ cells. It is likely that this population overlaps, at least partially, with the 20% of CD56^dim^ cells expressing the lower level of FcγRIIIa shown in Figure 1, which contained increased and decreased proportion of NKG2A^+^ and CD158a,h/b,j^+^ cells, respectively. On the other hand, the terminally differentiated CD57^+^ subset representing 30–60% cells has been shown to express a slightly higher (1.2-fold) level of FcγRIIIa as compared with the CD57^−^ subset and to contain a higher proportion of CD158^+^ cells and lower proportion of NKG2A^+^ cells (25, 26). In humans, a clonal or oligoclonal expansion of a subset of NK cells expressing or not the CD94-NKG2C receptor has been observed after human cytomegalovirus (HCMV) infection. These adaptive NK cells exhibit a surface receptor signature a mature phenotype (i.e., showing progressive gain of CD57 and KIRs and loss of NKG2A) and epigenetic remodeling. Furthermore, some adaptive NK cells display deficient expression of FcεRγ. These NK cells exhibit more robust IFN-γ and TNF production, but not degranulation after FcγRIIIa engagement. It is, however, of note that FcγRIIIa is equally expressed by conventional and adaptive NK cells. We also observed an association between FcγRIIIa, CD158a,h/b,j, and NKG2A expression. However, the association was a continuum rather than a positive/negative association: the greater the expression of FcγRIIIa on unstimulated CD56^dim^ cells, the greater and the lower the probability to coexpress CD158a,h/b,j and NKG2A, respectively (Figure 1B). Moreover, the magnitude of FcγRIIIa expression was substantial: the highest level of FcγRIIIa expression on CD56^dim^ NK cells from a given individual was 5- to 10-fold that of the lowest level (Figure 1A; Figure S1 in Supplementary Material). Therefore, the overexpression of FcγRIIIa we observed on CD158a,h/b,j^+^CD56^dim^ cells cannot be explained by the increased frequency of CD57^+^ cells observed within the latter population, indicating that it occurs before terminal differentiation marked by the acquisition of CD57. Thus, the gradual increase of FcγRIIIa expression is an important feature of the differentiation/maturation of CD56^dim^ cells, and we propose to use it as a new marker of this process.

A functional segregation related to CD56 expression has been previously reported: CD56^bright^ cells act mainly by secretion of cytokines, whereas CD56^dim^ cells are more prone to exert cytotoxicity (2, 3). CD56^bright^ cells respond primarily to monokine stimulation, whereas CD56^dim^ cells respond primarily to target cells (2, 5), so this functional dichotomy may depend mainly on the stimulation conditions. With CD56^dim^ cells, several reports have shown that IFN-γ^+^ and CD107a^+^ NK cells may be expressed in a mutually exclusive manner on simulation with different target cells including K562 cells (5, 7), P815 cells sensitized with anti-CD16 mAb (5), CD20^+^ cells sensitized with RTX or obinituzumab (17), *Plasmodium falciparum*-infected red blood cells (4) and *Drosophila* cells expressing several AR ligands (6). Our results, obtained in specific response to FcγRIIIa engagement by plate-bound anti-CD16 mAb or therapeutic mAbs or mAbs directed to ARs involved in natural cytotoxicity (i.e., in the absence of target cells), agree with these observations. However, Foley et al. observed a high frequency of CD107a-expressing cells not producing IFN-γ, an intermediate frequency of cells exhibiting both responses, and a low frequency of NK cells producing IFN-γ but not expressing CD107a (7). In our experiments, the subset that produced IFN-γ but did not degranulate usually dominated the subset of double-positive cells. This discrepancy may be related to the stimulation condition. In line with this is our observation that degranulating cells but not IFN-γ-producing cells from a given donors were substantially higher after stimulation with 3G8 than after stimulation with RTX or TTZ. The discrepancy may also result from our use of isolated NK cells vs thawed PBMCs in the previous study (7).

Fauriat et al. assumed that functional segregation may reflect differences in the kinetics of the responses (6). Indeed, in agreement with our results, degranulation occurred earlier than IFN-γ production. However, in our study, the dichotomy was observed as soon as IFN-γ production was detected and persisted for up to 20 h. This finding ruled out that the functional segregation we observed was related to the fact that cells that had already degranulated might produce IFN-γ later on. This conclusion is supported by our finding that the functional responses of CD56^dim^ NK cells were associated with different phenotypes: CD158a,h/b,j^+^ cells were more prone than NKG2A^+^ cells to produce IFN-γ in response to FcγRIIIa-dependent and FcγRIIIa-independent stimulation. These results are consistent with those reporting KIR expression associated with target cell-induced IFN-γ production by NK cells, but NKG2A was sufficient for degranulation (7, 25–27).

Distinct intracellular signaling pathways originating from different ARs lead to cytokine secretion or cytotoxicity (41–45). By contrast, the ability of one AR such as FcγRIIIa to preferentially induce one or the other response in individual NK cells demonstrates that a given pathway may lead to different responses in different NK cells from a given donor. Modest differences in MFI from donor to donor for any given AR may have a profound effect on the functional response to stimulation. IFN-γ production requires a higher level of activation than does degranulation (6). It may, therefore, be assumed that the high level of FcγRIIIa expression observed on CD158a,h/b,j^+^ cells, accounts for their tendency to preferentially produce cytokines in response to FcγRIIIa engagement. However, a clonal or oligoclonal expansion of a subset of NK cells expressing or not the CD94-NKG2C receptor has been observed after HCMV infection. These cells, which are called adaptive NK cells, exhibit a mature phenotype (NKG2A^−^CD57^+^KIRs^+^) and epigenetic remodeling. Although adaptive and conventional mature NK cells express equally FcγRIIIa, adaptive NK cells display more robust IFN-γ and TNF production, but not degranulation in response to FcγRIIIa engagement [recently reviewed in Ref. (46, 47)]. In addition, it has been shown that the ability of NK cells to produce IFN-γ in response to engagement of ARs involved in natural cytotocicity is gradually acquired and related to epigenetic remodeling of the *IFNG* promoter, during their terminal differentiation (27). It is, therefore, more likely that this epigenetic remodeling is a general feature of more differentiated cells and is responsible for the shift toward IFN secretion observed in the present study in response to FcγRIIIa engagement.

Finally, which function (killing or cytokine production) mediates clinical responses to therapeutic mAbs is not known. Among the most convincing evidence that ADCC plays a role in mediating the clinically relevant antitumor response to therapeutic mAbs is the demonstration by our group (31) and others (32–34) that *FCGR3A* gene polymorphism is associated with clinical responses to different cytolytic mAbs such as RTX (31–33), TTZ (34), and cetuximab (48). In accordance with this, an *in vitro* genotype–phenotype association has been observed: the *FCGR3A* polymorphism affects the concentration-effect association of rituximab-mediated ADCC by NK cells (37). These studies show that FcγRIIIa-expressing cells are involved in the mechanism of action of these mAbs, but they did not demonstrate which FcγRIIIa-expressing cells or which effector functions are involved in the *in vivo* situation. Although NK cells are considered to act through ADCC to mediate the mechanism of action of the different cytolytic mAbs, an indirect mechanism, whereby NK cells act by recruiting cells *via* FcγRIIIa-dependent cytokine production, remains possible. Following this hypothesis, functional heterogeneity related to the differentiation/maturation of CD56^dim^ NK cells could be involved in the variability of the clinical response observed in patients treated with cytolytic mAbs such as RTX or TTZ.

## Ethics Statement

Peripheral blood mononuclear cells were exclusively obtained from the blood of healthy volunteers (i.e., blood donors from the Etablissement français du Sang Centre-Atlantique, who had given their written informed consent) according to institutional research protection guidelines (Agreement No IMMUNOUMR6239/37/12/01).

## Author Contributions

LL, NC-J, and AB designed and performed the experiments. LL performed the statistical analysis. LL, NC-J, and GT analyzed the results. LL and GT wrote the manuscript, GT supervised the study conception and design. All authors critically revised the work, provided substantial input, and gave final approval to the version to be published.

## Conflict of Interest Statement

The authors declare that the research was conducted in the absence of any commercial or financial relationships that could be construed as a potential conflict of interest. The reviewer SV and handling editor declared their shared affiliation.

## Supplementary Material

Figure S1 Expression of Fc*γ*RIIIa on CD56^dim^ NK cells from seven donors. Freshly isolated NK cells were stained with fluorescent anti-CD56, anti-CD16, anti-NKG2A, anti-CD158a,h, and anti-CD158b,j mAbs for 30 min at 4ºC and analyzed by FCM for CD16 and CD56 expression. A first gate was set on CD16^+^CD56^dim^ NK cells (solid lines, similar whatever the donor). Five other gates (dotted white lines) were then set in order to divide CD16*+*CD56^dim^ NK cells into five equal parts (each gate containing 20% of the cells within the first gate). Given the substantial interindividual variation of Fc*γ*RIIIa staining, the setting of these five gates (position on the *x*-axis) differs from one donor to another. The percentage of NKG2A*+*, anti-CD158a,h*+*, and anti-CD158b,j*+* cells within each gate was then evaluated (Figure 1).
