# Supplementary figures and images for: Gradual Increase of FcγRIIIa/CD16a Expression and Shift toward IFN-γ Secretion during Differentiation of CD56dim Natural Killer Cells
Source: Front Immunol. 2017 Nov 20;8:1556. doi: 10.3389/fimmu.2017.01556 (PMC5701929; doi:10.3389/fimmu.2017.01556)

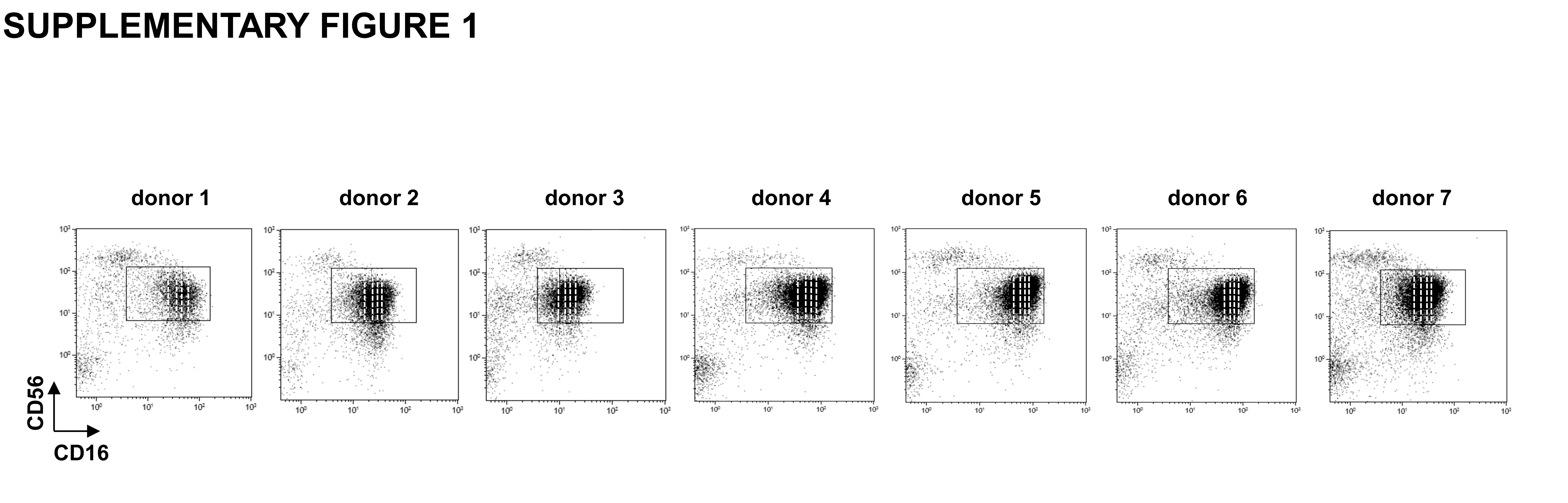

Supplement: Figure S1 — Expression of FcγRIIIa on CD56dim NK cells from seven donors. Freshly isolated NK cells were stained with fluorescent anti-CD56, anti-CD16, anti-NKG2A, anti-CD158a,h, and anti-CD158b,j mAbs for 30 min at 4ºC and analyzed by FCM for CD16 and CD56 expression. A first gate was set on CD16+CD56dim NK cells (solid lines, similar whatever the donor). Five other gates (dotted white lines) were then set in order to divide CD16+CD56dim NK cells into five equal parts (each gate containing 20% of the cells within the first gate). Given the substantial interindividual variation of FcγRIIIa staining, the setting of these five gates (position on the x-axis) differs from one donor to another. The percentage of NKG2A+, anti-CD158a,h+, and anti-CD158b,j+ cells within each gate was then evaluated (Figure 1). [file Image_1.tif]
